# p66Shc: A novel biomarker of tubular oxidative injury in patients with diabetic nephropathy

**DOI:** 10.1038/srep29302

**Published:** 2016-07-05

**Authors:** Xiaoxuan Xu, Xuejing Zhu, Mingming Ma, Yachun Han, Chun Hu, Shuguang Yuan, Yuan Yang, Li Xiao, Fuyou Liu, Yashpal S. Kanwar, Lin Sun

**Affiliations:** 1 Department of Nephrology, 2^nd^ Xiangya Hospital, Central South University, Changsha, Hunan, China; 2 Departments of Pathology & Medicine, Northwestern University, Chicago, USA

## Abstract

Increased p66Shc expression has been associated with diabetic nephropathy (DN). However, whether p66Shc can serve as a potential biomarker for tubular oxidative injury in DN is unknown. We measured the expression of p66Shc in peripheral blood monocytes (PBMs) and renal biopsy tissues from DN patients and then analysed the relationship between p66Shc expression and the clinical characteristics of patients with DN. Patients were divided into 4 groups (class IIa, class IIb, class III and the control group). qPCR, Western blotting and immunohistochemistry were performed. The results showed that both p66Shc and p-p66Shc expression significantly increased in PBMs and kidney tissues of DN patients. Moreover, Spearman’s correlation and multiple regression analyses were carried out. A positive relationship between the p66Shc expression and oxidative stress was found. p66Shc and oxidative stress were significant predictors of the degree of tubular damage. In addition, p66Shc expression was positively correlated with the concentrations of β-NAG, UACR and 8-OHdG, low-density lipoprotein and blood glucose levels, and duration of diabetes in patients with DN from class IIa to class III. These data indicated that increased expression of p66Shc may serve as a therapeutic target and a novel biomarker of DN.

Diabetic nephropathy (DN) is a severe microangiopathic complication in patients with both type 1 and type 2 diabetes mellitus. A number of risk factors have been associated with the progression of DN, including glomerular hypertension, proteinuria, hyperlipidaemia and genetic predisposition^1^. Studies carried out over the last 3 decades have indicated that there are a series of underlying mechanisms in the progression of kidney injury in DN. In recent years, excessive generation of reactive oxygen species (ROS) has emerged as the major pathogenetic denominator in the progression of DN^2^. During hyperglycaemia, excessive amounts of ROS are produced from both the NAPDH system and mitochondrial sources, leading to the formation of vascular lesions *via* metabolic modifications of target tissue molecules and disturbances in the intrarenal haemodynamics. As ROS induce renal injury, it is anticipated that renal tissue injury will be reflected in compromised renal functions^3^,^4^. Because ROS are the major inducers of renal injury in microvascular complications of diabetes, the molecules or the pathways involved in their generation could serve as therapeutic targets to ameliorate the progression of DN or alternatively could serve as biomarker(s) to monitor the progression of DN. Therefore, we explored the relevance of p66Shc in DN and determined whether it could serve as a biomarker during the progression of the renovascular complications of diabetes.

p66Shc is a member of the adaptor protein family, which is encoded by four loci in mammals. Three isoforms are encoded by ShcA, which include proteins with relative molecular weights of 46, 52 and 66 kDa. Among them, p46/p52 are ubiquitously distributed and are expressed in various tissues, while p66Shc has restricted tissue-specific expression^5^,^6^. All these proteins contain a phosphotyrosine binding domain (PTB), a collagen homology domain-1 (CH1) and a Src homology 2 domain (SH2)^6^. The p66Shc protein is distinct because it has an additional N-terminal region named CH2, which is responsible for its redox properties and is involved in lifespan regulation and apoptosis^7^. The structural features of Shc isoforms suggest that they play a role in diverse cellular functions; for example, p46Shc and p52Shc are involved in the transmission of mitogenic signals from tyrosine kinases to RAS proteins, while p66Shc is primarily associated with mitochondrial ROS production, oxidative stress and induction of apoptosis^5^. In addition, treatment of 293A cells (a human embryonic kidney cell line) with high glucose (HG) increased p66Shc expression, while there was no change in p46/p52 expression^8^. This suggests that p66Shc is specifically relevant to the pathogenesis of DN. In addition, several different studies have indicated that p66Shc is involved in various chronic diseases that are secondarily due to oxidative damage^9^,^10^,^11^,^12^. Furthermore, there are many *in vivo* and *in vitro* studies implicating p66Shc in the progression of DN *via* the modulation of mitochondrial ROS production, leading to oxidative stress in the kidney^13^,^14^. Our previous studies also suggest that expression of both p66Shc and the phosphorylated form of p66Shc (p-p66Shc) is increased in diabetic mouse models and associated with oxidative injury of the tubular cells of the kidney in DN^15^. Interestingly, genetic loss of the p66Shc gene in mice partially prevented glucose intolerance and premature death^16^. Moreover, Menini *et al*. demonstrated that mice with a p66Shc deletion showed less severe pathological renal lesions and lower proteinuria levels, glomerular sclerosis index and matrix accumulation under hyperglycaemic conditions^17^. Similarly, Pagnin *et al*. noted that diabetes induced p66Shc gene expression in circulating peripheral blood monocytes (PBMs) and demonstrated that the up-regulation of p66Shc was associated with increased oxidative stress^18^. Except for the studies on PBMs, very limited information is available on the status of p66Shc expression in patients with DN, and few reports have established a relationship between p66Shc expression and the clinical characteristics of patients with DN.

In view of the above considerations, we investigated the p66Shc expression in kidney tissues in patients with DN and established a correlation between p66Shc expression and the clinical characteristics of these patients. We noted that the p66Shc and p-p66Shc expression in PBMs as well as renal tissues was significantly increased in patients with DN compared to controls, and the up-regulated p66Shc and p-p66Shc expression was associated with increased ROS production, which was linked to cortical tubular lesions, suggesting that p66shc may serve as a new biomarker of tubular injury in patients with DN.

## Results

### P66Shc expression in patients with DN

The clinical characteristics of the DN patients and controls in this study are shown in Table 1. There were no significant differences between DN patients and controls in terms of age, sex, body mass index (BMI), alanine aminotransferase (ALT), aspartate aminotransferase (AST), high-density lipoprotein cholesterol (HDL-C), creatinine (Cr), systolic blood pressure (SBP), and diastolic blood pressure (DBP). Compared with the controls, the DN patients showed significantly increased levels of blood triglycerides, the estimated glomerular filtration rate (eGFR), low-density lipoprotein cholesterol (LDL-C), blood glucose, haemoglobin A1c (HbA_1C_), uric acid and 24 h urine protein excretion. In sharp contrast, serum albumin was dramatically decreased in DN patients. Interestingly, even in different DN groups, these physicochemical parameters are different. For example, the level of serum albumin was significantly decreased in class III versus class IIa and IIb. There were no significant differences in the other blood or urine parameters and in the clinical characteristics between the four groups.

**Figure 1:**
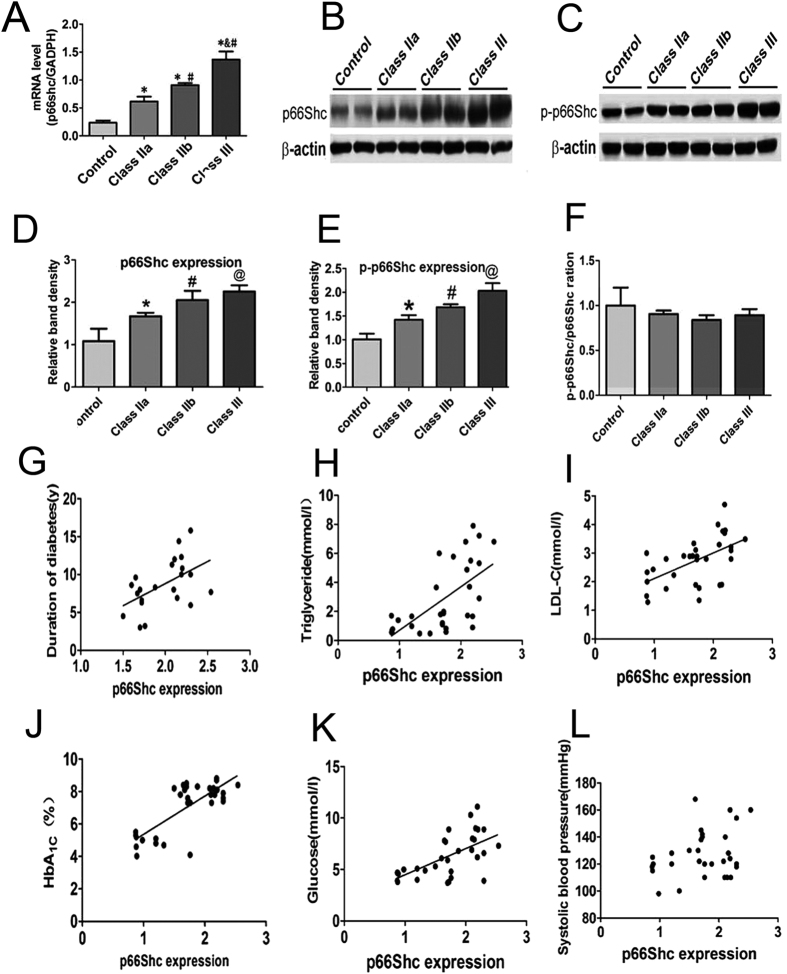
Expression of p66Shc mRNA and protein in PBMs and correlation analysis. p66Shc expression in the PBMs of DN patients was determined by real-time PCR analyses (**A**). Western blot analyses of p66Shc and p-p66Shc protein expression in PBMs (**B**,**C**). (**D**,**E**) Quantification of average band intensity of Western blots (Control: n = 36; Class IIa: n = 15; Class IIb: n = 18; Class III: n = 17). (**F)** Bar graph represents the ratio of p-p66Shc to p66Shc expression, as assessed by quantification of relative band intensity. Control: n = 36; Class IIa:n = 15; Class IIb:n = 18;Class III:n = 17. *p < 0.01 versus control; ^#^p < 0.05 versus class IIa; ^@^p < 0.01 versus IIb. Values are shown as the mean ± SD. Correlation analysis of the expression level of p66Shc in PBMs of patients with DN and duration of diabetes (**G**), triglycerides (**H**), LDL-C (**I**), HbA1C (**J**), blood glucose (**K**) and SBP (**L**). Values are means ± E.

As shown in Table 2 and Fig. 1B, the p66Shc protein level in PBMs of the DN groups was dramatically increased compared with the control group (p < 0.01). Between different DN groups, p66Shc mRNA levels in class IIb and class III were significantly higher than those of class IIa (p < 0.01). In addition, p66Shc mRNA levels in class III were higher than those of class IIb (Fig. 1A) (p < 0.01). The p66Shc and p-p66Shc protein levels were also increased in DN patients (Fig. 1B–E), while no significant differences in the ratio of p66Shc to p-p66Shc protein expression in each group was observed (Fig. 1F). To further analyse the p66Shc protein expression in PBMs of DN patients, a correlation analysis was carried out to assess whether there was an association with the development of DN. The results showed that the p66Shc protein expression was positively correlated with the duration of diabetes (Fig. 1G), levels of triglyceride (Fig. 1H), LDL-C (Fig. 1I), HbA1C (Fig. 1J) and blood glucose (Fig. 1K). No correlation was observed with the SBP (Fig. 1L). Similar results were also observed for p-p66Shc expression (Supplement Fig. 1A–F). The coefficient of correlation (R) of the above parameters is included in Table 2.

### Pathological lesions and the correlation between tubular interstitial damage and oxidative stress

**Figure 2:**
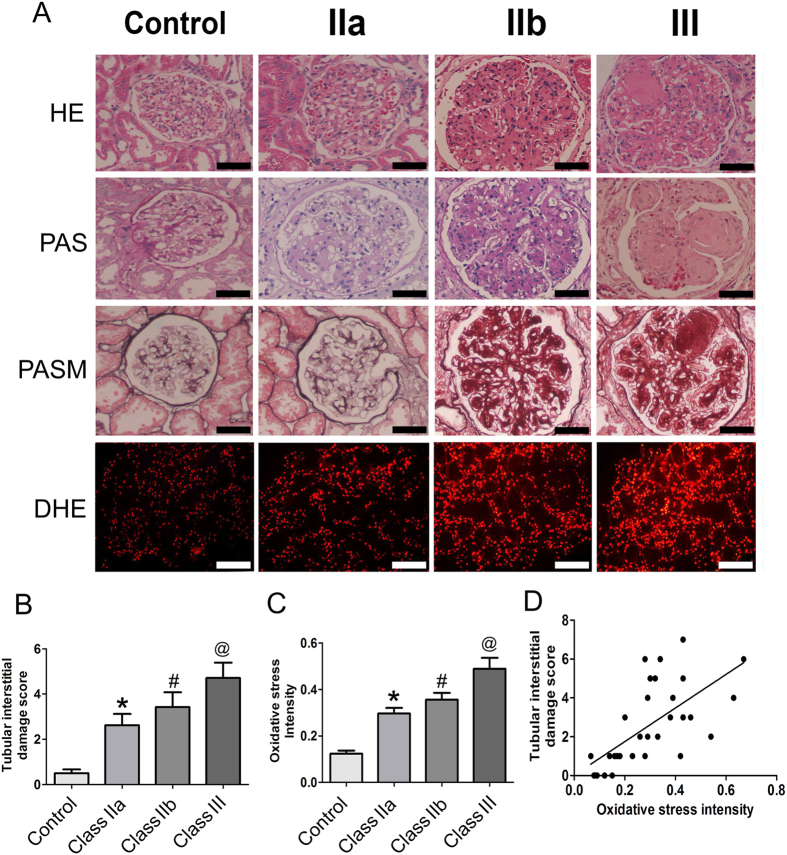
Pathological lesions and oxidative stress in the kidneys of DN patients and correlation analysis. (**A**) The histological changes in renal biopsies of DN patients were assessed by HE PAS and PASM staining. Scale bar with black color 50 μM (magnification ×200). DHE staining indicates the oxidative stress in kidney tissues Scale bar with white color 10 μM (magnification ×40). (**B)** The bar graphs represent tubular interstitial damage scores of the renal biopsies from DN patients and controls. (**C**) Fluorescence intensity of DHE staining reflects kidney oxidative stress. *p<0.01 versus control; ^#^p < 0.05 versus IIa; ^@^*p* < 0.01 versus IIb. (**D**) The analysis of the correlation between tubular interstitial damage score and kidney oxidative stress (r = 0.732, p < 0.01).

The morphological changes in the renal tissues of DN patients, including the glomerular and tubulointerstitial compartments, were assessed by HE, PAS and PASM staining. Light microscopy showed that samples from DN class II had mild mesangial expansion, while the mesangial expansion was more than 25% of the total mesangium in class III samples. Furthermore, Kimmelstiel-Wilson lesions were observed in class IIb and III tissues (Fig. 2A, upper panels). In addition, dihydroethidium (DHE) staining was performed in the sections from the renal biopsy of DN patients to measure oxidative stress, which is reflective of the ROS levels. Compared to the control group, significantly higher ROS levels were observed in the kidneys of patients with DN, which was accompanied by a higher degree of renal pathological changes (Fig. 2A, lower panels), and the increase in ROS levels positively correlated with renal pathological changes.

We then determined the tubulointerstitial damage score in the renal tubular compartment as previously described^19^. The total score for a 6 point scoring criteria in DN patients was determined. The control group had a score of 0-1 points, while scores of 1 to 4 points and 3 to 6 points were observed for class II and class III DN patients, respectively. Statistical analysis showed that there were significant differences between the DN groups and the control group and class IIa versus class III groups (p < 0.01), but no significant differences were found between class IIa versus IIb and class IIb versus III (Fig. 2B). Semiquantitative analysis demonstrated that oxidative stress intensity (ROS levels) in the kidneys of DN class III patients was significantly higher than that in class IIa and IIb, and there was no significant difference between class IIa and class IIb (Fig. 2C). Furthermore, the tubular interstitial damage score was positively correlated with the renal oxidative stress in DN patients (r = 0.653, p < 0.01) (Fig. 2D).

### p66Shc expression increased in the kidneys of DN patients and was correlated with oxidative stress

**Figure 3:**
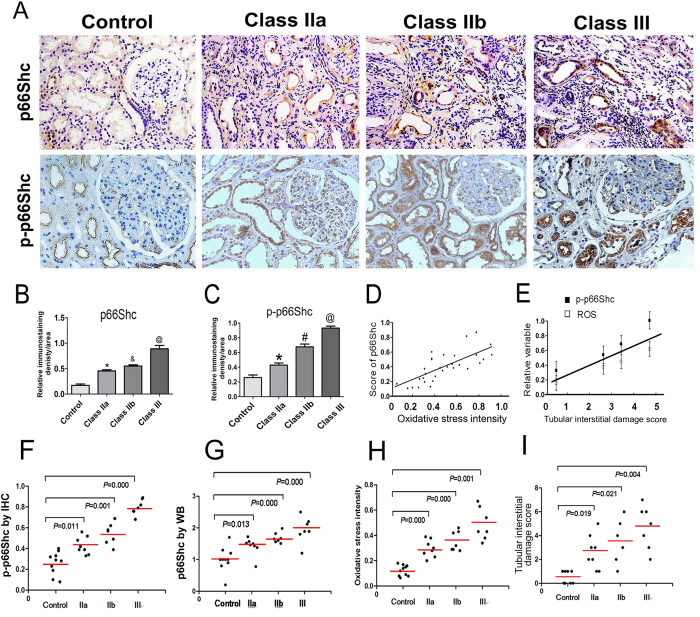
p66Shc expression in the kidneys of DN patients. (**A**) Immunostaining of p66Shc and p-p66Shc in renal biopsy tissues (magnification ×200). (**B**,**C**) IHC was performed. Relative immunostaining density of p66Shc and p-p66Shc in the kidney tissues of DN patients in each of the groups. *p < 0.01 versus control; ^#^p < 0.01 versus IIa; ^@^p < 0.01 versus class IIb. Values are shown as the mean ± SD. (**D**) Spearman’s correlation analyses showed the association between p66Shc expression by IHC and oxidative stress, as assessed by DHE staining. (**E**) Result of multiple regression model of showed the contribution of p66shc, oxidative stress to tubular injury score. (**F**–**I**) The levels of p-p66Shc by IHC and p66Shc expression by Western blot analysis, the degree of oxidative injury and tubular interstitial damage in kidney of DN patients were different compared to those of controls on different groups of pathological classification (IIa to III) in patients with DN.

p66Shc protein expression in the kidneys of patients with DN was assessed by immunohistochemical (IHC) staining with mouse monoclonal anti-p66Shc and anti-phospho-p66Shc (Ser36) antibodies. IHC staining revealed a dramatic increase in p66Shc and p-p66Shc expression in DN patients compared with the control group (p < 0.01). The increase was predominantly confined to the renal proximal tubules, and very little expression was observed in the glomerular mesangium (Fig. 3A). Further analysis revealed that the expression of p66Shc and p-p66Shc was accompanied by an increase in severity of renal lesions in DN patients, and expression up-regulated in the class IIb and class III groups (Fig. 3B,C). In addition, correlation analyses showed a positive relationship between p-p66Shc expression and the relative ROS levels (r = 0.774) (Fig. 3D). Multiple regression analysis found a significant relative correlation among p66Shc, oxidative stress and tubular oxidative damage (Fig. 3E). The regression coefficient p-p66Shc (β1) contributed to tubular oxidative injury is 5.100, P = 0.007.The regression coefficient ROS (β2) contributed to tubular oxidative injury is 3.364, P = 0.041 (Fig. 3E). Furthermore, for analyzing the levels of p66Shc and p-p66Shc expression in different groups of pathological classification (IIa to III) in DN patients by the ANOVA analysis. A significant difference compared to control was found. Further investigation showed a significant difference was also seen on the degree of oxidative damage or tubular interstitial damage in the different pathological classification groups (Fig. 3G–I).

### p66Shc expression was positively correlated with tubular damage and various clinical characteristics in DN patients

**Figure 4:**
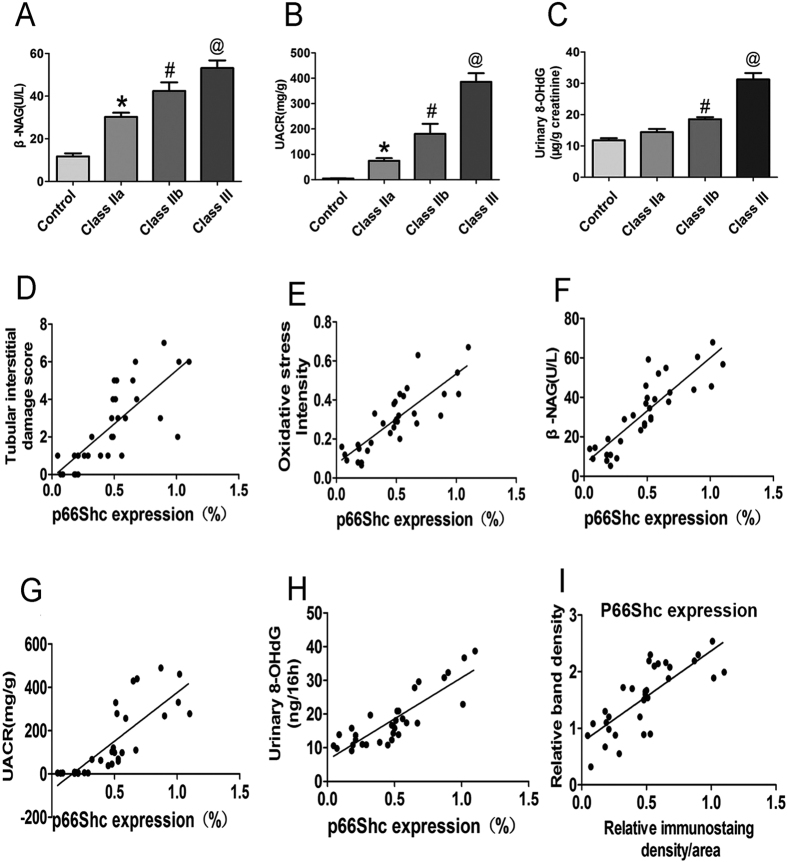
Correlation of p66Shc expression in renal tissues and clinical parameters. (**A)** Bar graph represents the levels of urine β-NAG in patients with DN in each group. (**B**) The concentration of urine albumin: creatinine ratio (UACR) in patients with DN in each group. (**C**) Levels of urine 8-OHdG in each group. (**D**) Correlation analysis indicated that p66Shc expression in renal tissues of DN patients and the tubular interstitial damage score were positively correlated (r = 0.762, p < 0.01). (**E**) The p66Shc expression levels and the kidney oxidative stress were also positively correlated (r = 0.812, p < 0.01). (**F**) A positive correlation was found between the expression of p66Shc and urine β-NAG level (r = 0.812, p < 0.01). Correlations were also found for the concentration of UACR (**G**) (r = 0.830, p < 0.01) and urine 8-OHdG levels (**H**) (r = 0.874, p < 0.01) and p66Shc expression. The analysis of the correlation between the expression of p66Shc in the kidneys and PBMs is shown (**I**) (r = 0.632, p < 0.01). Values are shown as the mean ± SD. r: correlation coefficient.

To test the relationship between p66Shc expression and tubular injury in DN patients, we first assessed the tubular damage in each group by measuring the urine levels of N-acetyl-β-glucosaminidase (β-NAG), urine albumin-to-creatinine ratio (UACR) and 8-OHdG in each group. The results showed that in the DN groups, the concentration of β-NAG, UACR and 8-OHdG increased with severity of the disease from DN class IIa to class III (Fig. 4A–C). Further analyses demonstrated that p66Shc expression in the kidneys of DN patients was positively correlated with tubulointerstitial damage (Fig. 4D), renal oxidative damage degree (ROS level) as detected by DHE staining (Fig. 4E), and urinary levels of β-NAG (Fig. 4F) and UACR (Fig. 4G). In addition, the linear regression analysis demonstrated that p66Shc and p-p66Shc expression in the kidneys of DN patients was positively correlated with the level of urinary 8-OHdG (Fig. 4H). Furthermore, analysis also revealed that p66Shc expression in the kidney and PBMs in DN patients were positively correlated (Fig. 4I). In the other hand, similar results were observed in the correlation of p-p66Shc expression in the kidney and the clinical indicators in DN patients (Supplement Fig. 2A–F).

## Discussion

Excessive intracellular ROS generation in diabetes is considered a common pathway in hyperglycaemia-induced renal injury. The oxidative stress in diabetes patients is relatively high compared to healthy individuals, and it increases proportionately with the severity of the disease^20^. p66Shc, an oxidative stress response protein, is known to modulate mitochondrial ROS production. However, the relationship between p66Shc expression and the clinical characteristics of patients with DN had not been explored. This study reports three major findings. First, p66Shc expression was found to be increased in both PBMs and kidney tissues of DN patients. Second, there was a good correlation between the increased expression of p66Shc protein in the kidney tissues and in the PBMs of DN patients with renal oxidative stress. Third, we noted that p66Shc expression was positively correlated with tubular injury in patients with DN. These findings suggested that the increased expression of p66Shc may contribute to the progression of DN in patients with diabetes, and p66Shc in PBMs may serve as a potential biomarker to monitor the progression of DN in the future.

Recent studies have indicated that increased p66Shc expression is associated with ROS generation in human aortic endothelial cells exposed to high glucose (HG) conditions^8^. Other reports found that inhibition of p66Shc can reduce superoxide generation, maintain endothelial homoeostasis and alleviate kidney damage in diabetes and HG conditions^13^. However, it is unclear whether measuring the expression of oxidative stress-related adaptor proteins, such as p66Shc in the PBMs of DN patients, can serve as a marker of kidney damage. Urine 8-OHdG concentration may serve as an oxidative stress biomarker in various kidney diseases, but it lacks sensitivity and specificity for the early stages of DN. In this regard, DHE staining may serve as a marker for oxidative stress damage in renal biopsy tissues of DN patients, but it may not be practical because collecting kidney specimens during the early stages of DN could be a major impediment. Interestingly, expression of p66Shc in the PBMs and kidneys of patients with DN both had a positive correlation with the protein expression of p66Shc and p-p66Shc, tubular damage and oxidative stress. Likewise, enhanced p66Shc mRNA expression in the PBMs was associated with oxidative stress, indicating that measurement of p66Shc mRNA or p66Shc protein expression in PBMs may be a simple and a feasible potential biomarker for kidney oxidative stress and may be an important index gauging the progression of DN in diabetic patients.

In the present study, we also noted that the expression of p66Shc in PBMs and renal tissues of DN patients was positively correlated with the duration of diabetes, levels of triglycerides, HbA1C, LDL-C and blood glucose and tubular interstitial damage and renal oxidative stress (Figs 1, 2, 3 and Table 2). It is known that diabetes of long duration, poor glycaemic control, hypertension, and hyperlipidaemia are significant risk factors for the progression of DN^21^. Undoubtedly, HbA1C level is an indicator of chronic hyperglycaemia in diabetic kidney disease, and the clinical presentation has a strong relationship with oxidative stress in diabetic conditions^22^,^23^. Similarly, it is known that LDL-C levels are modified by oxidative stress in hyperglycaemic conditions^24^,^25^. Kim *et al*. found increased expression of p66Shc by LDL via alteration of CpG hypomethylation^26^. In addition, oxidized LDL (oxLDL) promoted p66Shc phosphorylation, while knockout of p66Shc decreased oxLDL-induced ROS generation and prevented the oxidative damage in endothelial cells^27^. These data indicate that the p66Shc expression in the PBMs may be a predictive marker of the progression of renal oxidative damage in DN patients.

Analysis of the relationship between tubular interstitial damage and renal oxidative stress indicated that the tubular interstitial injury score was increased in different categories of DN (Fig. 2). Previously, *in vivo* and *in vitro* studies in our laboratory demonstrated that the p66Shc expression was confined to the proximal tubular cells, and its expression was up-regulated in HK-2 cells (a human proximal tubular cell line) when exposed to high-glucose conditions and angiotensin II^15^. In the present study, we also found that the p66Shc and p-p66Shc were predominantly expressed in the renal tubular cells, and little expression was observed in the glomerular mesangium in renal biopsy tissues from DN patients. The expression of p66Shc and p-p66Shc was significantly increased in kidneys of DN class IIa, IIb and III. In addition, the increased p66Shc expression in kidneys was positively correlated with the degree of tubular damage and the levels of urinary β-NAG, 8-OHdG and UACR (Figs 3, 4), suggesting that renal tubular oxidative injury is probably modulated by p66Shc during the progression of DN. On the other hand, we further proved that there were a notable correlation between p66Shc expression and relative ROS level in kidney tissues of DN patients (Fig. 3D) or p-p66Shc, oxidative injury (ROS levels) and tubular interstitial damage, respectively (Fig. 3E). In addition, by ANOVA analysis, compared to control, a significant difference were observed on p-p66Shc expression in pathological classification IIa, II b and III of DN patients, respectively, Also similarly results was found in that of p66Shc expression, oxidative damage and tubular interstitial damage (Fig. 3F–I). This date indicated that the tubular oxidative damage in kidney of DN patients was associated with increased p66Shc or p-p66Shc expression.

Renal tubular damage plays a key role in the pathogenesis of DN, which may be independent of glomerular lesions^28^,^29^. Moreover, early tubular proliferation induced by oxidative stress in hyperglycaemic environments may contribute to the glomerular hyperfiltration^30^; thus, we also examined the relationship between p-p66Shc expression and renal functions. Unfortunately, there was no positive correlation (Table 2), which suggested that we may need to increase the sample size in future studies. Certainly, the p66Shc expression in PBMs was positively correlated with renal injury (Fig. 4). Therefore, it is possible that the PBMs with increased p-p66Shc levels invaded the kidneys, resulting in renal oxidative injury by locally generated ROS in the kidney tissues.

The mechanisms that mediate up-regulation of p66Shc expression and its phosphorylation in DN are not fully understood. However, the epigenetic regulation of p66Shc transcription is well known^31^. Cross-talk between p53 and p66Shc was observed in experimental models of diabetes^32^, in which p53 binds to the putative p53 binding sequence in the p66Shc promoter and then induces p66Shc transcription^33^. In addition, Sirt1 also binds to the p66Shc promoter, which could directly inhibit p66Shc transcriptional activity through epigenetic chromatin modification^8^. Furthermore, Sirt1 also inhibits p53 transcriptional activity induced by HG, which may affect the p66Shc expression^34^. In addition, activated protein C exerts its antioxidant effects by reversing glucose-induced hypomethylation and hyperacetylation of the p66Shc promoter^5^, suggesting that both p53 and Sirt1 are key regulators of p66Shc gene transcription. However, activated p53 and decreased Sirt1 expression have been found in DN *in vivo* and *in vitro*. Increased expression of p53 was found in the kidneys of STZ-treated rats and db/db diabetic mice^35^. OxLDL may trigger the phosphorylation and activation of p53, as demonstrated in HK-2 cells^36^. Additionally, it is known that HG directly attenuates the deacetylase activity of Sirt1 in HK-2 cells^37^. Thus, activation of p66Shc transcription in PBMs and the kidney tissues of DN patients may be a result of up-regulation of p53 and decreased activity of Sirt1. The next question that needs to be addressed is the mechanism underlying the increased phosphorylation of p66Shc in DN patients. It is well known that PKC-β induces phosphorylation of p66Shc38, and HG increases the synthesis of ANG II39. Our previous studies indicated that HG and Ang II could activate PKC-βand PKC-δ, which then phosphorylate p66Shc at the Ser36 residue. The phosphorylated p66Shc thus becomes a target for the prolyl isomerase Pin1, which recognizes the proline residue following the phosphorylated serine residue. After phosphorylation, p66Shc translocates into the mitochondria, and ROS generation is increased, leading to tubular oxidative injury^15^,^40^. In addition, ERK has been shown to phosphorylate p66Shc at Ser36 in tubular cells^41^, and ET-1 can also induce MEK/ERK-dependent p66Shc serine phosphorylation^42^, indicating that many of these above events might participate in phosphorylation of p66Shc, resulting in tubular oxidative injury in patients with DN.

Taken together, our study shows that increased expression of p66Shc may play a prominent role in renal tubular oxidative injury in the progression of DN. Moreover, p66Shc expression in PBMs may serve as a potential biomarker to monitor DN progression. Our study also indicates that inhibition of p66Shc may be a novel therapeutic strategy in the amelioration of renal tubular injury in DN.

## Materials and Methods

### Participants

Fifty patients with DN and thirty-six non-DN controls (minimal change disease) were recruited with written consent. All procedures were carried out in accordance with the approved guidelines. The DN patients were categorized into class IIa, IIb and III according to the 2010 pathologic classification of DN^43^. Class I and class IV were excluded because they have mild or nonspecific light microscopy changes or present with advanced diabetic glomerulosclerosis. The class IIa group included 15 patients (8 males and 7 females), aged 32–48 years (mean: 46.25 ± 8.53 years); the class IIb group included 18 patients (9 males and 9 females), aged 33–56 years (mean: 44.29 ± 9.250 years); and the class III group included 17 patients (8 males and 9 females), 42–58 years (mean: 51.14 ± 5.047 years). The control group included 36 patients (18 male and 18 females), aged 32–58 years (mean: 42.2 ± 8.066 years). Patients were diagnosed in the Second Xiangya Hospital, Central South University, according to the World Health Organization diagnostic criteria for type 2 diabetes. Patients did not use adrenal cortical hormones or immunosuppression drugs. The institutional review board and the administrators of the Department of Nephrology in Second Xiangya Hospital approved the protocol for this study. Informed consent was obtained from all the participants.

### Biochemical analysis of blood and urine

The liver and renal functions, serum triglycerides, total cholesterol (TC), high-density lipoprotein cholesterol (HDL-C), low-density lipoprotein cholesterol (LDL-C) as well as blood glucose levels were analysed using standard automated enzymatic methods (Hitachi 912 automated analyser). N-acetyl-β-D-glucosaminidase (β-NAG) was determined by an automated colorimetric method (Pacific Biomarkers, Inc.). The concentration of 8-hydroxy-2′-deoxyguanosine (8-OHdG), a critical biomarker of oxidative stress and DNA damage in urine, was measured by ELISA (8-OHdG Check, Nikken Foods, Fukuroi, Shizuoka, Japan) by following the instructions provided by the vendor^44^.

### Peripheral blood monocyte preparation

Peripheral blood (6 ml) was collected from various patients and then mixed with 6 ml of Dulbecco’s phosphate-buffered saline (PBS). The samples were centrifuged at 2,000 rpm for 30 min for separation into different layers. The turbid white layer containing the mononuclear blood cells was collected, rinsed with PBS twice and centrifuged at 1,500 rpm for 10 min. The mononuclear cells were then incubated in RPMI 1640 culture medium (Gibco BRL, Grand Island, USA) with 10% foetal calf serum (Invitrogen, Carlsbad, CA) at 37 °C in a 5% CO_2_ atmosphere. The adherent cells were collected and subjected to flow cytometry following staining with a CD-14 monoclonal antibody (Santa Cruz Biotechnology Inc., Texas, USA).

### Quantitative real-time polymerase chain reaction

Total RNAs of the PBMs were prepared using TRIzol (Invitrogen). First-strand cDNAs were prepared by two-step RT-PCR (Fermentas Life Science). Relative gene expression was determined using SYBR Green quantitative real-time PCR assays on a 7500 Fast Real-Time PCR System (Applied Biosystems, Carlsbad, CA). The cycling conditions were as follows: denaturing at 50 °C for 2 min, 95 °C for 10 min, then 40 cycles of 95 °C for 30 sec and 60 °C for 60 sec, followed by 72 °C for 1 min. All reactions were carried out in triplicate with a non-template control. The specific primers for p66Shc were as follows: forward, 5′-GCCGAGTATGTCGCCTA TGT-3′; reverse, 5′-GGGTGGGTTCCTGAGGTATT-3′. The primers for GADPH were as follows: forward, 5′-AGAAGGCTGGGGCTCATTTG-3′; reverse: 5′-AGGGGCCATCCACAGTCTTC-3′.

### Western blotting assay

PBMs from DN patients (class IIa, class IIb, class III) and controls were prepared using radio immunoprecipitation assay (RIPA) buffer containing protease inhibitors. Protein concentration was measured with a Pierce^®^ BCA Protein Assay Kit (Pierce Inc., USA). Thirty micrograms of total protein from each sample was subjected to electrophoresis and then transferred onto a PVDF membrane. The expression of p66Shc was assessed using a mouse monoclonal IgG anti-Shc/p66 (N-terminus) from LifeSpan BioSciences (1:1000 dilution). This antibody reacts with the p66Shc protein and not with the p46/p52 isoforms. The p-p66Shc protein expression was assessed using anti-Shc (phospho S36) (1:1000 dilution; Abcam). This antibody only recognizes the 66 kDa form of the Shc protein that is phosphorylated (p-p66Shc66 at Ser36), and it does not cross-react with unrelated phosphorylation sites in the protein or with the non-phosphorylated form of other Shc proteins. β–actin (1:6000 dilution) was used as an internal loading control. The ratio of p-p66Shc to actin and p66Shc to p-p66Shc was quantified using the Kodak 1D image analysis system.

### Morphological analysis of kidneys

Four μm thick paraffin sections of a renal biopsy were stained with haematoxylin-eosin (H & E), periodic acid Schiff (PAS) and Masson. Tubular injury was scored as previously described^19^,^45^.

### Immunohistochemistry

Renal biopsy tissue was paraffin-embedded and sectioned. After de-paraffinization, the sections were incubated with 3% H_2_O_2_ solution to block the endogenous peroxidase. Antigen retrieval was carried out using EDTA solution (pH 9.0) for 10 min. Sections were incubated with mouse monoclonal IgG anti-Shc/p66 (N-terminus) and anti-phospho-p66Shc (Ser36). They were incubated overnight at 4 °C, followed by incubation with horseradish peroxidase-conjugated secondary antibody and diaminobenzidine (DAB) substrate sequentially^19^,^44^,^45^. After haematoxylin counterstaining and dehydration, the sections were mounted and analysed with a Nikon microscope.

### Statistical analysis

Statistical analyses were performed using SPSS 19.0 software. Data are expressed as the mean ± SD. Correlation analyses were carried out using Pearson’s correlation, Spearman’s correlation analysis and multiple regression analyses. p < 0.05 was considered statistically significant.

## Additional Information

**How to cite this article**: Xu, X. *et al*. p66Shc: A novel biomarker of tubular oxidative injury in patients with diabetic nephropathy. *Sci. Rep.* **6**, 29302; doi: 10.1038/srep29302 (2016).

## Supplementary Material

Supplementary Figure

## Figures and Tables

**Table 1:** Clinical Characteristics of the Patients.

	Control (n = 20)	Class IIa (n = 11)	Class IIb (n = 10)	Class III (n = 11)
Age(y)	42.2 ± 8.066	46.25 ± 8.53	44.29 ± 9.25	51.14 ± 5.047
Sex(m/f)	15/5	8/3	7/3	8/3
Course of disease(y)	–	6.0 ± 2.2	8.9 ± 1.7	12.8 ± 2.3
BMI(kg/m^2^)	23 ± 3.5	23.9 ± 2.3	24.8 ± 3.2	24.9 ± 4.1
ALT(IU/L)	18.3 ± 4.2	20.2 ± 3.5	17.8 ± 5.6	20.4 ± 3.8
AST(IU/L)	24.2 ± 9.2	23.9 ± 8.2	25 ± 7.5	24.7 ± 6.3
Alb(g/l)	38.9 ± 3.9	32.8 ± 2.3^1^	30.2 ± 1.8^2,3^	29.5 ± 3.4^2,3,4^
Total cholesterol (mmol/l)	4.0 ± 1.1	4.1 ± 1.2	4.2 ± 2.1^1^	4.3 ± 1.8^1^
Triglycerides (mmol/l)	0.9 ± 0.4	1.58 ± 1.01^2^	4.15 ± 1.8^2,3^	5.4 ± 2.6^2^
HDL-cholesterol (mmol/l)	1.5 ± 0.4	1.6 ± 0.6	1.8 ± 0.9	2.3 ± 0.4
LDL-cholesterol(mmol/l)	2.1 ± 0.6	2.7 ± 0.6^1^	2.8 ± 0.9^1^	3.6 ± 0.6^2,3^
Creatinine (mg/dl)	0.7 ± 0.2	0.8 ± 0.1	0.8 ± 0.6	0.9 ± 0.4
eGFR (ml/min/1.73m^2^)	80.1 ± 12	72.3 ± 10^2^	70.1 ± 8.9^2^	68.3 ± 7.8^2^
BUN(mg/dl)	12.8 ± 2.3	13.7 ± 3.4	14.1 ± 6.2^1^	17.1 ± 7.2^2^
Glucose(mmol/l)	4.5 ± 0.5	5.8 ± 1.8^1^	7.1 ± 0.7^2^	8.5 ± 2.4^2^
HbA_1C_(%)	4.8 ± 0.5	7.9 ± 0.5^2^	8.0 ± 0.5^2^	8.1 ± 0.4^2^
Uric acid (umol/l)	350 ± 68	402 ± 87^1^	412 ± 90^2^	430 ± 110^2^
24h urine protein	–	2.4 ± 1.8^2^	3.3 ± 1.9^2^	4.9 ± 3.7^2^
SBP(mmHg)	108 ± 9.0	110 ± 3.5	113 ± 4.3	120 ± 6.2
DBP(mmHg)	75 ± 11	80 ± 9.2	82 ± 7.4	84 ± 10.3

Abbreviations: y, years; m, male; f, female; BMI: body mass index; ALT, alanine aminotransferase; AST, aspartate aminotransferase; Alb, albumin; HDL, high-density lipoprotein; LDL-cholesterol, low-density lipoprotein; eGFR: estimated glomerular filtration rate; BUN: blood urea nitrogen; HBA1C, glycosylated hemoglobin; SBP, systolic blood pressure; DBP, diastolic blood pressure; ^1^P < 0.05 versus control; ^2^P < 0.01 versus control; ^3^P < 0.05 versus IIa; ^4^P < 0.05 versus IIb. Values are means ± E.

**Table 2:** Correlate of the expression of p66shc and p-p66Shc protein in PBMs and some parameters by univariate analysis.

	p-p66Shc		p66Shc	
	r	P	r	P
Age (y)	0.308	0.086	0.212	0.076
Course of disease (y)	0.544	<0.01	0.512	<0.05
BMI (kg/m^2^)	0.045	0.520	0.032	0.076
ALT (IU/L)	0.033	0.480	0.087	0.065
AST (IU/L)	0.044	0.779	0.054	0.432
Alb (g/l)	0.092	0.557	0.098	0.355
Total cholesterol (mmol/l)	0.062	0.694	0.356	0.231
Triglycerides (mmol/l)	0.583	<0.01	0.639	<0.01
HDL-cholesterol (mmol/l)	0.209	0.897	0.377	0.078
LDL-cholesterol (mmol/l)	0.41	<0.01	0.552	<0.01
Creatinine (mg/dl)	0.256	0.535	0.298	0.387
eGFR (ml/min/1.73m^2^)	0.120	0.444	0.412	0.465
BUN (mg/dl)	0.263	0.089	0.352	0.132
Glucose (mmol/l)	0.499	<0.01	0.619	<0.01
HbA_1C_ (%)	0.645	<0.01	0.768	<0.01
Uric acid (umol/l)	0.078	0.367	0.192	0.254
24h urine protein	0.109	0.890	0.342	0.65
SBP (mmHg)	0.220	0.225	0.330	0.063
DBP (mmHg)	0.055	0.325	0.410	0.23

Abbreviations are as shown in Table 1.
