# Author Correction: p66Shc: A novel biomarker of tubular oxidative injury in patients with diabetic nephropathy

**DOI:** 10.1038/s41598-020-62787-1

**Published:** 2020-04-08

**Authors:** Xiaoxuan Xu, Xuejing Zhu, Mingming Ma, Yachun Han, Chun Hu, Shuguang Yuan, Yuan Yang, Li Xiao, Fuyou Liu, Yashpal S. Kanwar, Lin Sun

**Affiliations:** 1 Department of Nephrology, 2nd Xiangya Hospital, Central South University, Changsha, Hunan China; 2 Departments of Pathology & Medicine, Northwestern University, Chicago, USA; 3 Present Address: Health Management Center, Xiangya Hospital, Central South University, Changsha, Hunan China

Correction to: *Scientific Reports* 10.1038/srep29302, published online 05 July 2016

In Figure 2A, the panels showing the DHE staining for the control, IIa, IIb and III classes are incorrect. The correct Figure 2 appears below as Figure 1.

**Figure 1:**
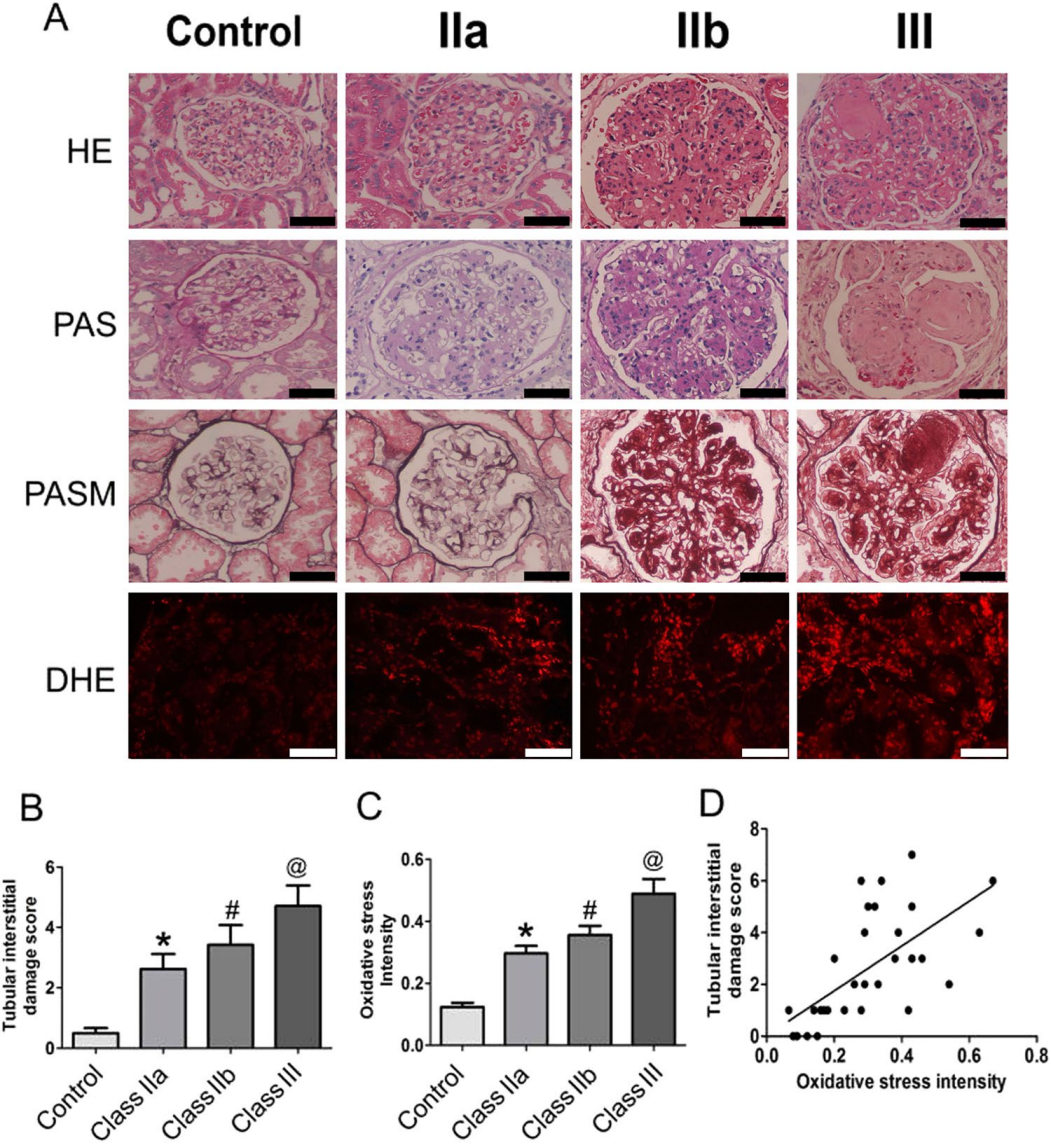
.

