# Supplementary material for: p66Shc: A novel biomarker of tubular oxidative injury in patients with diabetic nephropathy
Source: Sci Rep. 2016 Jul 5;6:29302. doi: 10.1038/srep29302 (PMC4932503; doi:10.1038/srep29302)
Supplement: Supplementary Figure [file srep29302-s1.pdf]

# p66Shc: A novel biomarker of tubular oxidative injury in patients with diabetic nephropathy

XiaoxuanXu[1, 3], Xuejing Zhu[1], Mingming Ma[1], Yachun Han[1], Chun Hu[1], Shuguang Yuan[1], Yuan Yang[1], Li Xiao[1], Fuyou Liu[1], Yashpal S. Kanwar[2], Lin Sun[1]

XiaoxuanXu and Xuejing Zhu contributed equally to this work.

[1]Department of Nephrology, 2nd Xiangya Hospital, Central South University, Changsha, Hunan, China;

[2]Departments of Pathology & Medicine, Northwestern University, Chicago, USA

[3]Present address: Health Management Center, Xiangya Hospital, Central South University, Changsha, Hunan, China

***Running title:*** p66Shc expression in diabetic nephropathy patients

***Key words:*** p66Shc, Diabetic nephropathy, ROS, renal pathology, tubulo-interstitial lesions

***Correspondence address:***

Lin SUN, MD, Ph.D
Department of Nephrology
Second Xiangya Hospital
Central South University
NO 139,Remin Road
Changsha, Hunan, P.R.China, 415800
Tel: 86-731-8529-2064
E-mail: zndxsunlin11@163.com

**Suppl. Fig 1**

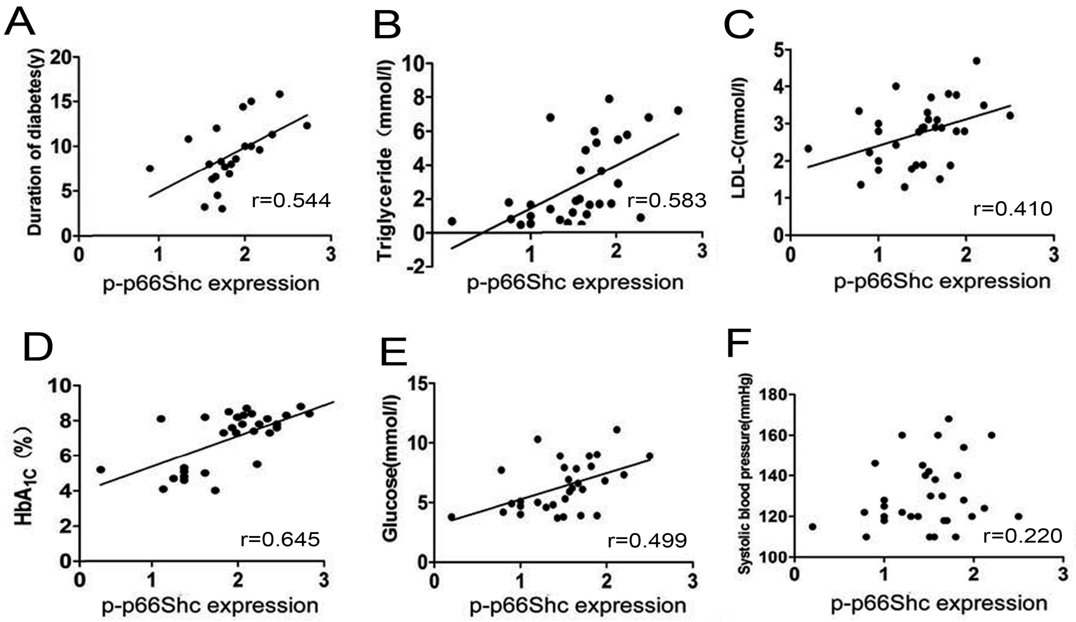
A
Duration of diabetes(y)
r=0.544
p-p66Shc expression
B
Triglyceride (mmol/l)
r=0.583
p-p66Shc expression
C
LDL-C(mmol/l)
r=0.410
p-p66Shc expression
D
HbA1C (%)
r=0.645
p-p66Shc expression
E
Glucose(mmol/l)
r=0.499
p-p66Shc expression
F
Systolic blood pressure(mmHg)
r=0.220
p-p66Shc expression

**Suppl. Fig 2**

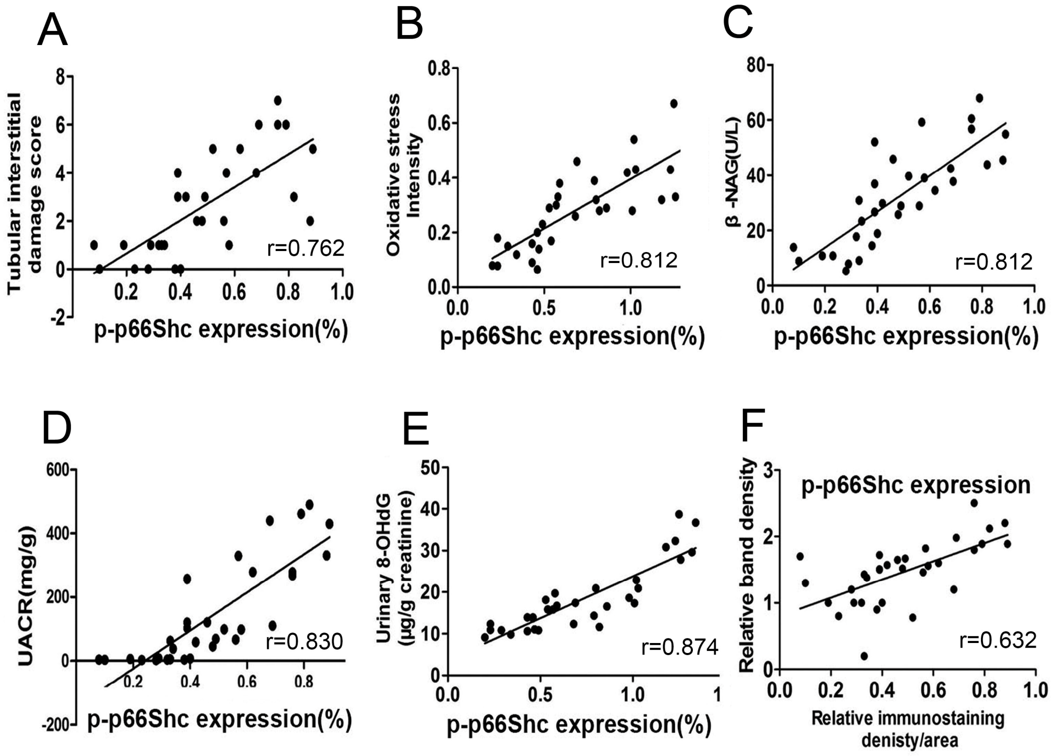


## Supplementary figure legends

**Suppl. Fig 1. The correlation analysis between phosphorylation of p66Shc (p-pShc) expression levels in PBM and various clinical characteristics in DN patients** . A. Correlation analysis between the expression level of p66Shc in PBMs of patients with DN and duration of diabetes(A)(r=0.544,p<0.01), triglyceride (B) (r=0.583, p<0.01), LDL-C (C) (r=0.410, p<0.01), HbA1C (D) (r=0.645, p<0.01), blood glucose (E) (r=0.499, p<0.01) and SBP (F) (r=0.220, p>0.05). Values are means ± E. r: Correlation coefficient.

**Suppl. Fig 2. The correlation of p-p66Shc expression in the kidney and the clinical indicators in DN patients**. A. Correlative analysis revealed that the p-p66Shc expression in renal tissues of DN patients and tubular interstitial damage score were positively correlated ($r=0.762$, $p<0.01$). B.The p-p66Shc expression levels and the kidney oxidative stress were also positively correlated ($r=0.812$, $P<0.01$). C. positive correlation was observed in the expression of p66Shc and urine β-NAG level ($r=0.812$, $P<0.01$). Likewise was case for the concentration of UACR (D) and urine 8-OHdG levels (E) and the p66Shc expression ($r=0.830$, $P<0.01$, $r=0.874$, $P<0.01$, respectively). F. The analysis of the correlation between the expression p66Shc in kidney and PBMs is shown ($r=0.632$, $P<0.01$). Values are means ± E. r: Correlation coefficient.
